# Recalibrating the body: visuotactile ventriloquism aftereffect

**DOI:** 10.7717/peerj.4504

**Published:** 2018-03-15

**Authors:** Majed Samad, Ladan Shams

**Affiliations:** 1 Department of Psychology, University of California, Los Angeles, CA, United States of America; 2 Department of Bioengineering, University of California, Los Angeles, CA, United States of America

**Keywords:** Multisensory, Recalibration, Visuotactile, Ventriloquism, Perception

## Abstract

Visuotactile ventriloquism is a recently reported effect showing that somatotopic tactile representations (namely, representation of location along the surface of one’s arm) can be biased by simultaneous presentation of a visual stimulus in a spatial localization task along the surface of the skin. Here we investigated whether the exposure to discrepancy between tactile and visual stimuli on the skin can induce lasting changes in the somatotopic representations of space. We conducted an experiment investigating this question by asking participants to perform a localization task that included unisensory and bisensory trials, before and after exposure to spatially discrepant visuotactile stimuli. Participants localized brief flashes of light and brief vibrations that were presented along the surface of their forearms, and were presented either individually (unisensory conditions) or were presented simultaneously at the same location or different locations. We then compared the localization of tactile stimuli in unisensory tactile conditions before and after the exposure to discrepant bisensory stimuli. After exposure, participants exhibited a shift in their tactile localizations in the direction of the visual stimulus that was presented during the exposure block. These results demonstrate that the somatotopic spatial representations are capable of rapidly recalibrating after a very brief exposure to visually discrepant stimuli.

## Introduction

The nervous system is at all times playing a guessing game with the aim of identifying which sensations should be integrated and which ought to be segregated. For example, consider what happens when a sound and sight are concurrently processed by the brain. If they originate from different sources, but the brain erroneously infers that they have a common cause, this can lead to an illusion known as the ventriloquist illusion, wherein the perceived location of the sound is captured by the location of the visual stimulus (Alais & Burr, 2004; Bischoff et al., 2007; Howard & Templeton, 1966). A similar phenomenon has been shown to occur between auditory and tactile representations (Caclin et al., 2002). However, when some of the sensations involve somatosensation, these guesses may also lead to aberrant bodily percepts, such as the rubber hand illusion (Botvinick & Cohen, 1998; Armel & Ramachandran, 2003; Samad, Chung & Shams, 2015). Moreover, we have previously shown that visual and tactile stimuli interact in somatotopic coordinates such that estimations of tactile stimulus location are biased towards concurrently presented visual stimuli (Samad & Shams, 2016).

Much work on the audiovisual ventriloquist illusion over the past several decades has revealed its independence from the direction of visual attention (Bertelson et al., 2000; Vroomen, Bertelson & Gelder, 2001) and that its effects are spatially specific to the trained region encoded in a hybrid receptive field that is a mixture of head-centered and eye-centered (Kopčo et al., 2009).

In the audiovisual ventriloquist illusion, prior work has uncovered evidence that there is an accompanying aftereffect that develops as a result of exposure to spatially discrepant audiovisual stimulus pairs such that subsequent auditory localizations are biased towards the position of the visual stimulus (Lewald, 2002; Recanzone, 1998) and moreover that it emerges very rapidly (Frissen, Vroomen & Gelder, 2012), and can also occur in the temporal domain when the discrepancy is an asynchrony (Burg, Orchard-Mills & Alais, 2015; Van der Burg, Alais & Cass, 2015; Orchard-Mills, Van der Burg & Alais, 2016). In brief, this effect is interpreted as a recalibration of the mapping between auditory and visual spatial representations. In a study that examined this, participants were given an exposure phase where they were presented with audiovisual stimulus pairs that always had a constant disparity between them for no longer than 10 min (Wozny & Shams, 2011). Results from that study showed that participants’ localizations after this exposure were significantly biased in the direction of the visual stimulus that was paired with the auditory stimulus during this exposure phase (Wozny & Shams, 2011).

While this kind of spatial recalibration has been shown for auditory and visual spaces, it is not yet clear whether the somatotopic space is similarly malleable. We designed an experiment to test the hypothesis that visuotactile ventriloquism induces an aftereffect such that prolonged exposure to spatially incongruent visuotactile stimuli results in a measurable recalibration of tactile representations. Given that our previous study (Samad & Shams, 2016) identified a vigorous interaction between visual and tactile stimuli in the somatotopic space, we hypothesized that an aftereffect would also be observable such that tactile representations would be biased, dependent on the disparity between the visuotactile stimuli that were presented during the exposure phase.

## Method

### Participants

Thirty-seven individuals (23 female) with a mean age of 21.3 gave written consent to participate for course credit. All participants had normal or corrected-to-normal vision. The experimental methods were approved by the UCLA IRB. One participant was excluded from the experiment for non-compliance with instructions. The remaining participants (*N* = 36) were randomly assigned to two groups, Vision-Left (*n* = 18) and Vision-Right (*n* = 18).

### Stimuli and apparatus

We used a the same setup that was described in Samad & Shams (2016). It comprised three components: a tactor array that will be described further below, a ceiling mounted projector that was redirected downward onto participants’ forearms via a 45° angled mirror, and a computer that was running Matlab with PsychToolBox for stimulus presentation.

**Figure 1:**
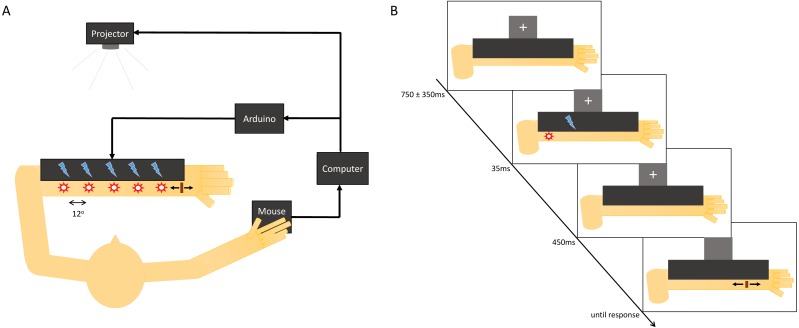
Experimental setup and localization trial design. (A) Diagram depicting the experimental setup used to present the stimuli, including a tactor array that is driven by a microcontroller (Arduino) for tactile stimulus delivery, and a projector for visual stimulus delivery, and a computer for control of both. (B) The sequence for a localization trial: a fixation cross is presented on the screen for a variable interstimulus interval, after which stimuli are presented for 35 ms. After a 450 ms interval, the fixation cross disappears and a cursor appears for the participant to make responses with.

The tactor array was composed of a soft foam material measuring 30.5 by 10 cm^2^, in which five tactors were embedded (Pico Vibe 9 mm vibration motors −25 mm type; model number 307–103; Precision Microdrives, London, UK), spaced apart 41.1 mm, a distance that subtended 12° of visual angle. Thus the five locations were −24°, −12°, 0°, 12°, 24° with respect to fixation. The tactors were driven by a custom-built controller circuit that used an Arduino (Arduino, Salerno, Italy) to interface with MATLAB. The foam block was mounted on a piece of acrylic of the same dimensions that was fixed to the tabletop with the use of a hinge joint that allowed the block to be pressed onto participants’ forearms. This was aided by a 750 mL opaque bottle filled to a weight of ∼2 kg, that was used to ensure a complete contact of the tactors with the forearm. The fixation cross was displayed on a cardboard square (affixed to the bottle) such that it appeared 30° above the central tactor position. The visual stimulus was a white disk of light subtending 1.5°, presented by the projector at a location that was 30° below fixation, at one of five points coinciding with the positions of the tactors. Care was taken to ensure that each participant placed their forearm into the setup such that half of the forearm lengthwise was under the foam block, and would thus feel the vibrotactile stimuli, and the other half would be exposed to the projector’s screen and would thus have the visual stimuli displayed directly upon it. This enabled the bisensory stimuli to be as proximate to one another while allowing for both to be presented directly on the surface of the arm (see Fig. 1A). Note that this apparatus was designed for use with participants’ left forearms, so that they may use the computer mouse to register their responses with their right hands.

Additionally, Gaussian white noise at ∼70 dB was used to mask the audibility of the tactors by being played on headphones worn by the participants simultaneously with stimulus presentation. The volume was determined in pilot experiments such that the location of the tactile stimuli could not be determined on the basis of the tactor noise alone. Participants had their head position fixed by means of a chin-rest that was placed 195 mm away from the tactor array. Participants were allowed to adjust the height of the chair and/or the chinrest to achieve a comfortable posture.

### Procedure

**Figure 2:**
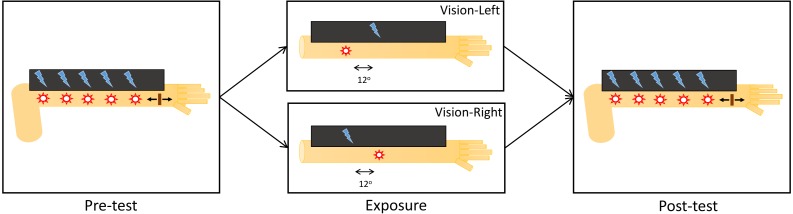
Experimental design. During pre- and post-test blocks, participants performed a visuotactile localization task. During the exposure block, participants had to passively attend to visuotactile pairs that were spatially discrepant such that the visual stimulus was either 12° displaced towards the elbow (Vision-Left) or towards the wrist (Vision-Right) with respect to the tactile stimulus.

The experiment consisted of three blocks: pre-test, exposure, and post-test. The total duration of the experiment was 2 h, and all three blocks ran consecutively in the same session. During pre- and post-test blocks, participants localized visual and tactile stimuli delivered to their arm in both unisensory and bisensory conditions that were randomly interleaved. The post-test block contained some top-up exposure trials interleaved. During the exposure block, participants were exposed to visual-tactile stimulus pairs that were always spatially incongruent and with a constant disparity between them (±12°). In order to familiarize participants with the task they were to perform, we included 15 trials of practice before the pre-test and exposure blocks. See Fig. 2 for a schematic of the experimental design.

The pre-test block consisted of 420 trials and took about 35 min to complete. Every possible pairing of visual-tactile positions was repeated 12 times in a pseudorandomized order (5 × 5 = 25 bisensory trial types), and we also interleaved 12 repetitions of each of the unisensory stimulus positions (5 + 5 = 10 unisensory trial types). Participants were to report the location of the stimuli presented, using a mouse cursor that spanned the same space where the visual stimuli were presented. The color of the cursor indicated to the participant which stimulus to respond to, blue for visual stimuli and red for tactile stimuli. The order of appearance of these two cursors was counterbalanced across participants.

Each trial started with the presentation of a fixation cross 30 degrees above the middle stimulus position, and was followed by the stimulus that was on the screen for 35 ms. The fixation cross was taken off the screen and the cursor appeared at a random horizontal location spanning the stimulus space to eliminate any biasing effects of a consistent starting location 450 ms after the stimulus offsets. Participants moved the cursor using a Bluetooth wireless mouse and were instructed to “move the cursor as quickly and accurately as possible to the position where you saw/felt the stimulus and click the left mouse button”.

The exposure block consisted of 40 trials and took approximately 10 min to complete. On every trial, a train of 20 successive spatially incongruent stimulus pairs were presented to participants at the same location. The disparity was such that the visual stimulus was displaced by 12° towards the elbow for the Vision-Left group and by 12 degrees towards the wrist for the Vision-Right group. A uniform distribution was used to select a random pair between the 5th and the 15th pairs that would be presented with the visual stimulus 100% brighter than on other pairs. Participants were instructed to click the left mouse button when they saw the brighter stimulus. Upon a successful detection, the stimulus pair changed position whilst maintaining the same spatial disparity between the visual and the tactile stimuli, and the same train of stimuli was presented, with a newly selected random pair to be presented with the brighter visual stimulus, until the next successful detection. Failures to detect the brighter visual stimuli caused the train of stimulus pairs to repeat until a brighter stimulus was detected.

Finally, participants performed 420 trials of spatial localization with 90 top-up trials interleaved (in segments with nine trials each) during the post-test block. The localization trials were identical to those performed in the pre-test block and the top-up trials were identical to those performed in the exposure block. Nine top-up trials were performed after every 40 localization trials had been completed. The post-test block took a total of 45 min to complete. The participants were instructed to perform the localization task during the localization trials and to perform the brightness detection task while passively observing the stimuli during the top-up trials.

On 10% of all trials, the fixation cross changed color to red simultaneously with stimulus presentation and participants were asked to report when this change occurred by clicking the right mouse button, which advanced them to the next trial. This was done to ensure that participants were fixating during stimulus presentation.

Participants were given the opportunity to take a 1–2 min break after every 150 localization trials, for a total of five breaks, two in the pre-test block and three in the post-test block. Pilot experiments determined that arm fatigue resulting from keeping it static and outstretched in the apparatus necessitated frequent breaks. Breaks never took place during the exposure block or during the top-up trials in the post-test block.

## Results

Firstly, to ensure that our participants fixated during the experiment, we calculated the proportion of catch trials on which participants failed to click the right mouse button—indicating that they had not seen the fixation cross change color. On average participants failed to detect 1% of these catch trials. This high performance in the fixation task indicated that participants indeed followed the instructions and fixated on the fixation point in the vast majority.

**Figure 3:**
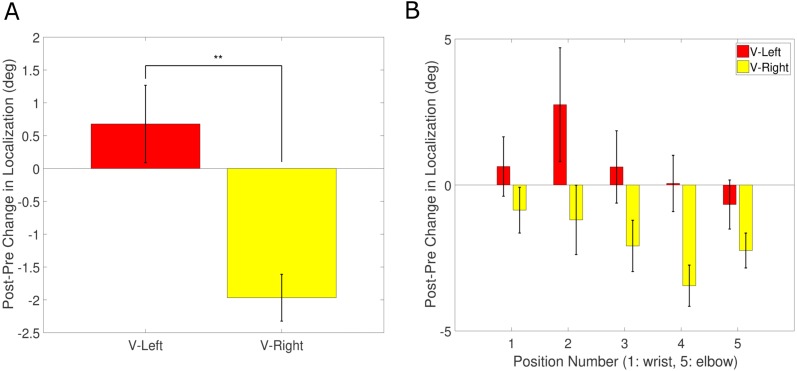
Unisensory tactile recalibration results. (A) Average change in localization of unisensory tactile stimuli from pre-test to post-test, collapsed across stimulus position, for both groups of participants. A positive shift indicates a shift towards the elbow, which is in the direction where the visual stimulus was presented with respect to the tactile stimulus during the exposure block for the Vision-Left group. (B) Change in localization of unisensory tactile stimuli from pre-test to post-test as a function of stimulus position, for both groups of participants. ** indicates statistical significance at *p* < 0.01. Error bars represent S.E.M.

We first analyzed the effects of exposure on unimodal tactile localizations, by computing the difference between the tactile localization on tactile-only trials in the pre-test and the post-test blocks. These differences were coded so that they are positive when the tactile localization is shifted towards the elbow, which is where the visual stimulus would have been presented for the Vision-Left group. Thus, we expect a positive shift for the Vision-Left group and a negative shift for the Vision-Right group. These results are shown in Fig. 3.

We analyzed the difference between tactile localizations on tactile-only trials from pre-test to post-test using an ANOVA with between-subjects factor “group” (two levels: Vision-Left and Vision-Right) and within-subjects factor “tactile-position” (five levels: −24, −12, 0, 12, 24). This analysis revealed a significant effect of “group”, *F*(1, 34) = 7.41, *p* = .01, and a trend for “tactile-position”, *F*(4, 136) = 2.36, *p* = .057. Therefore, in the absence of a strong effect of stimulus position, we collapsed this data across positions and computed one-sample t-tests comparing the change from pre- to post-test to zero. The Vision-Left group did indeed exhibit a positive shift on average (*M*. = 0.68, *S.E.M.* = 0.83), though not significantly different from zero. Conversely, the Vision-Right group exhibited a negative shift (*M*. =  − 1.97, *S*.*E*.*M*. = 0.50), which was found to be significantly different from zero, *t*(17) =  − 3.91, *p* = .001, Cohen’s *d* = 0.92. To look at whether the two groups differed in this change, and because we had a strong hypothesis driven reason to expect that the Vision-Left group pre- to post-test difference ought to be greater than that for the Vision-Right group we computed a one-tailed independent samples *t*-test on this data across positions comparing the Vision-Left group to the Vision-Right group, *t*(34) = 2.72, *p* = .005, Cohen’s *d* = 0.91 (see Fig. 3). This analysis therefore shows that there was a statistically significant effect of exposure condition on the shift in the unisensory tactile localizations from pre-test to post-test such that participants who were exposed to the Vision-Left condition exhibited errors in tactile localization further to the left than those exposed to the Vision-Right condition.

Next, we investigated whether this effect was also present in the bimodal trials. We again analyzed the difference between tactile localizations on bimodal trials from pre-test to post-test using an ANOVA with between-subjects factor “group”, within-subjects factor “visual-position” (five levels: −24, −12, 0, 12, 24) and within-subjects factor “tactile-position” (five levels: −24, −12, 0, 12, 24). The results of this analysis again revealed only a significant effect of “group”, *F*(1, 34) = 8.38, *p* < .01. Collapsing across both visual and tactile positions, and then running a one-tailed independent samples *t*-test comparing the Vision-Left group (*M*. = 0.7, *S*.*E*.*M*. = 0.86) to the Vision-Right group (*M*. =  − 2.08, *S*.*E*.*M*. = 0.44) showed a statistically significant difference, *t*(34) = 2.90, *p* < .01, Cohen’s *d* = 0.97.

Finally, we also analyzed the visual-only trials for any effect of the exposure condition. We similarly analyzed this data using an ANOVA with between-subjects factor “group”, and within-subjects factor “visual-position” (five levels: −24, −12, 0, 12, 24). The results of this analysis revealed a significant main effect of “group”, *F*(1, 34) = 4.41, *p* < .05, as well as a significant main effect of “visual-position”, *F*(4, 136) = 2.98, *p* < .05. Post-hoc tests showed that only localization changes for “visual-position” 0 as compared to 24 survived Bonferroni correction for multiple comparison, *t*(34) =  − 3.1, *p* < .05, and that the Vision-Left group exhibited on average a negative shift (*M*. =  − 0.342, *S*.*E*.*M*. = 0.145), which was significantly different from zero, *t*(17) =  − 2.36, *p* < .05, Cohen’s *d* = 0.56, whereas the Vision-Right group exhibited on average a positive shift ( *M*. = 0.217, *S*.*E*.*M*. = 0.223), which was not found to be significantly different from zero. In both cases, this shift was in the direction of the tactile stimulus that was presented during the exposure block.

## Discussion

Results demonstrate that participants who were briefly exposed to synchronous but spatially incongruent visual-tactile pairs of stimuli exhibited a subsequent bias in their localizations of tactile stimuli presented in isolation, which corresponded to the direction of the visual stimuli that they were exposed to. This represents a visual-tactile ventriloquism aftereffect that closely parallels the audiovisual ventriloquism aftereffect (Alais & Burr, 2004; Wozny & Shams, 2011; Lewald, 2002; Recanzone, 1998). This demonstrates the generality of the rules of integration and plasticity throughout the nervous system, and that the rapid recalibration of sensory maps to each other is not restricted to exteroceptive modalities but is also an actively utilized process in the mapping between the somatotopic and visual representational spaces.

Interestingly, we also observed a change in visual localization behavior as a result of the exposure block. Participants in the Vision-Left group had visual localization biases after the exposure that were in the direction of the exposed tactile stimuli. Importantly, this bias was approximately one order of magnitude smaller than the bias that was observed for tactile localization.

These results are consistent with recent findings demonstrating cross-modal plasticity in adulthood, a finding that emerges from studies investigating the effect of visual deprivation on the haptic modality (Kauffman, Théoret & Pascual-Leone, 2002; Merabet et al., 2008; Lo Verde, Morrone & Lunghi, 2016). In those studies, the emphasis is placed on the fact that an unused modality can lead to its cortical machinery being used to aid task performance in another modality. Here, we extend that idea by showing that cross-modal plasticity may also operate when the spatial mapping between one modality and the other is recalibrated. We posit that this causes a change in the recalibrated modality’s unisensory representation as demonstrated by the fact that the change in tactile-only trials was dissociated across our two groups (see Fig. 3A).

Ventriloquism as measured by localization paradigms has often been criticized for its susceptibility to response bias (Choe et al., 1975; Vroomen & Stekelenburg, 2014). Paradigms making use of aftereffects are generally more robust to response bias due to the aftereffect being measured on unisensory trials, i.e., in the absence of the cross-modal stimulus that might otherwise bring about the response bias. Nevertheless, it remains a possibility that the aftereffect may represent response learning, rather than a true change in the sensory representation. That said, in the present study, participants were not asked to make localization responses during the exposure block, minimizing the possibility that a response bias might have been learned and would have persisted into the post-test block.

In conclusion, we have demonstrated that visual-somatotopic spatial recalibration occurs after very brief exposure to spatially discrepant stimuli and therefore that the somatotopic space is more malleable than was previously thought.

## Supplemental Information

10.7717/peerj.4504/supp-1 Supplemental Information 1 Tactile Localization Database This archive contains 20 .csv files for the tactile unisensory localization errors in the pre-test and the post-test from the subjects in the groups designated as “VT” and “TV”. The files are labelled as for example “vt_pre_1” indicating that the data corresponds to the pre-test tactile localization errors for the “VT” group from position number 1 (position 1 is nearest to the wrist and position 5 is nearest to the elbow). Each column represents a subject, and rows are individual trials.

## Additional Information and Declarations

### Data Availability

The following information was supplied regarding data availability: The raw data has been supplied as a Supplementary File.
